# Complementary feeding practices and nutritional status in infants living in Turkey: Iowa infant feeding attitude scale and complementary feeding index

**DOI:** 10.1111/mcn.13746

**Published:** 2024-10-21

**Authors:** Bilge Meral Koc, Tugce Ozlu Karahan, Ezgi Arslan Yuksel, Gokcen Garipoglu

**Affiliations:** ^1^ Department of Nutrition and Dietetics Ihlamur Yildiz Cad. Faculty of Health Sciences, Bahcesehir University Istanbul Turkey; ^2^ Department of Nutrition and Dietetics Beyoglu Faculty of Health Sciences Istanbul Bilgi University Istanbul Turkey

**Keywords:** complementary feeding, infant growth, infant nutrition

## Abstract

Although complementary feeding (CF) and maternal attitudes towards infant feeding are known to affect the nutritional status of infants during the neonatal period, studies in this field remain limited. The present study aimed to determine CF practices for infants aged 6–12 months who live in Turkey and maternal attitudes towards infant feeding. In addition, the effects of CF practices and maternal attitudes on the nutritional status of infants were examined. This study included 720 infants, of whom 289 were aged 6–8 months and 431 were aged 9–12 months. CF status was assessed using the CF index (CFI), and maternal attitudes towards infant feeding were measured using the Iowa infant feeding attitude scale (IIFAS). The weight and length measurements of infants were categorized using z‐scores. Infants with weight‐for‐age z‐score (WAZ) and weight‐for‐length z‐score (WLZ) of less than −2 standard deviation were categorized as malnourished. Mothers of malnourished infants were found to have lower CFI and IIFAS scores (*p* < 0.05). Consistency of fruit feeding and complementary foods in the first 3 days of CF was associated with WAZ. The mothers' level of knowledge about CF and appropriate CF practices significantly affects the nutritional status of infants. The tools used in the present study to monitor CF practices should be incorporated into public health programmes.

## Key messages

- The mothers' level of knowledge about complementary feeding and appropriate practices significantly affects the nutritional status of infants.
- Mothers of malnourished infants were found to have lower complementary feeding practices.
- The mode of delivery was found to affect both weight‐for‐age and weight‐for‐length z‐scores of infants.
- Fruit feeding for the first 3 days affected weight‐for‐age z‐scores negatively.

## Graphical abstract

The study examined complementary feeding (CF) practices and maternal attitudes towards infant feeding in Turkey, involving 720 infants aged 6–12 months. Results showed lower CF and maternal attitude scores in mothers of malnourished infants, highlighting the need for public health programmes to improve CF knowledge and practices to enhance infant nutrition.

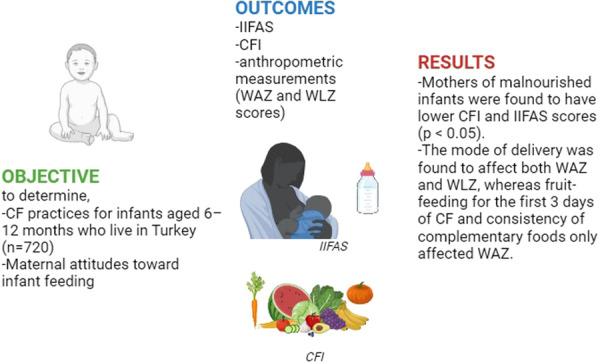

## 1 INTRODUCTION

Breast milk is essential for infants' healthy growth and development (Eksioğlu et al., 2016). The World Health Organization (WHO) and the United Nations International Children's Emergency Fund recommend that infants should be exclusively breastfed for the first 6 months after birth and should start receiving complementary foods at 6 months, with continued breastfeeding up to 2 years of age (World Health Organization, 2003). However, the duration of breastfeeding remains suboptimal worldwide, including in Turkey. In developing countries, only 39% of infants aged <6 months have been reported to be breastfed. Moreover, according to the report published by the Turkey Demographic and Health Survey (TDHS), the proportion of infants exclusively breastfed during the first 4–5 months was only 14% (HUIPS, 2018). WHO reports that the most appropriate time for complementary feeding (CF) is the 180th day, and consultancy is provided under the guidance of WHO in Turkey. However, the rate of starting complementary feeding at the appropriate time in Turkey is 37% for infants < 6 months (HUIPS, 2018). Another issue that is as important as the time to start complementary feeding is which foods to begin with. It has been reported that the food to be given in the first days should be from the vegetable group. However, it has been reported that the first foods given to infants in Turkey when starting complementary feeding are yogurt and cheese, as well as vegetables (TDHS, 2018). Some gaps should be considered when switching to complementary feeding in addition to breast milk. Adequate intake of nutrients, such as energy, protein, iron and vitamin A should be ensured. These deficiencies, especially protein, can negatively affect growth and development (Fewtrell et al., 2017).

Attitudes towards nutrition when starting complementary feeding during the period when breastfeeding is discontinued have an impact on the growth and development of infants. It has been reported that suboptimal complementary feeding negatively affects z‐scores, which are important criteria in the growth status of infants (Clayton et al., 2024). Globally, child malnutrition remains a major public health problem (Zongrone et al., 2012). When breast milk alone is no longer sufficient to meet all nutritional requirements of the infant, especially after 6 months, complementary foods should be initiated to ensure optimal growth and development (World Health Organization, 2003). Complementary feeding is an important stage in the transition from breast milk to solid foods. According to the TDHS report, 85% of infants consume solid, semisolid, or soft foods at 6–8 months (HUIPS, 2018). In fact, the timing of the transition to CF has been associated with malnutrition in most infants (Fewtrell et al., 2017). A study from Turkey revealed that initiation of CF before 6 months of age was associated with inadequate energy and nutrient intake. In contrast, delayed initiation of CF was found to be associated with delayed growth (Doğan et al., 2019).

Maternal attitudes, intentions and behaviours play a major role in infant feeding (Atkinson et al., 2021). Studies investigating mothers' perspectives and decision‐making about breastfeeding have revealed that maternal attitudes towards breastfeeding affect infant feeding practices (Karande & Perkar, 2014; Yu et al., 2020). In fact, maternal attitudes towards feeding have been demonstrated to influence the decision regarding whether to breastfeed or formula‐feed an infant (Cox et al., 2015). Initiation and continuation of breastfeeding are associated with maternal age, educational level, socioeconomic status and the presence of supportive people (Yu et al., 2020). Mothers who have a positive attitude towards breastfeeding are more likely to engage in more positive practices of continued breastfeeding and infant nutrition (Akin et al., 2021). In this regard, Owais et al. (2019) reported that mothers with positive attitudes towards CF were less likely to initiate CF precociously.

Previous studies have revealed a relationship between infant and child feeding practices, as recommended by WHO, and child growth (Zongrone et al., 2012). However, few studies have investigated the impact of maternal attitudes and behaviours towards infant feeding on the nutritional status of infants. This study aimed to (1) determine CF practices for infants aged 6–12 months; (2) examine the effects of CF and breastfeeding on the nutritional status of infants; and (3) evaluate maternal attitudes towards infant feeding.

## 2 METHODS

### 2.1 Study design

Data for this cross‐sectional study were collected using a Google survey form between February and May 2023 from mothers of infants aged 6–12 months. Parents with infants aged 6–12 months were recruited through official announcements on the researchers' social media platforms. Participants could complete the questionnaire only once for one infant. Before the online survey, information was provided about the study, the voluntariness of participation and the possibility of withdrawing from participation at any stage of the survey without saving responses. Consent and eligibility were determined using checkboxes that had to be filled in before participants were allowed to take the survey. All data from the online form were collected anonymously. The sample consisted of 777 mothers with an infant or infants aged 6–12 months living in Turkey. Exclusion criteria included women with preterm birth or a chronic disease that may affect the growth and development of infants. The power of the study was determined using the G*Power software suite (G*Power 3.1.9.2, Duesseldorf, Germany). A power analysis was performed for sample selection, which yielded a type 1 error of α = 0.05, a type 2 error of β = 0.20 and a test power of 1 (β = 0.80). Calculations were performed in line with a similar study (Faul et al., 2007), based on a 95% confidence interval (alpha = 0.05) and 95% power, which yielded a minimum sample size of 255 participants. Mothers with infants aged 6–12 months were included in the study based on criteria, such as willingness to participate in the study, not feeding their infants exclusively with breast milk, not having twins and not having congenital anomalies or diseases that may affect the growth and development of their infants. We categorized infants into two groups: 6–8 and 9–12 months, according to psychomotor development; 6–8 months: Proprioceptive‐auditory play, babbling; 9–12 months: Appearance of syllable, repetitive babbling and imitation of adult speech (Sampallo‐Pedroza et al., 2015). A survey containing questions regarding sociodemographic characteristics of mothers and infants, anthropometric measurements of infants, breastfeeding and some CF practices was completed via an online platform. Maternal attitudes towards CF were measured using the Iowa Infant Feeding Attitude Scale (IIFAS), whereas CF practices were assessed using the CF index (CFI) scoring system.

### 2.2 Nutritional status

The weight and length measurements of infants as reported by the mothers were used to calculate the weight‐for‐age z‐score (WAZ), length‐for‐age z‐score (LAZ) and weight‐for‐length z‐score (WLZ). z‐scores were calculated for each infant using WHO Anthro software (Anthroplus WHO, 2009). The z‐score system allows the measurement of all three indices, presenting the results in terms of z‐scores against median values of an international reference population derived from anthropometric data (Seetharaman et al., 2007). Infants whose z‐scores are below the reference median value by 2 standard deviations (i.e. a z‐score below −2) are considered malnourished, stunted or underweight. Infants whose z‐scores are over the reference median value by 2 standard deviations (i.e. a z‐score: +2 standard deviations ‐ +3 standard deviations) are considered overweight. Infants whose z‐scores are over the reference median value by 3 standard deviations (i.e. a z‐score over +3) are considered obese. These categories; wasting or malnourished (low weight‐for‐height), stunted (low height‐for‐age) and underweight (low weight‐for‐age), overweight and obese (high weight‐for‐age). Cutoff values for z‐scores were determined based on the WHO cutoff values (WHO, 2006).

### 2.3 IIFAS

The IIFAS, developed by De La Mora and Russell in 1999, was designed to analyse maternal attitudes towards breastfeeding and infant feeding practices. The IIFAS is a reliable measurement tool to identify mothers at risk of early breastfeeding cessation and assess the attitudes of partners/relatives in various field studies (Mora et al., 1999). The Turkish version of the scale was analysed for validity and reliability. The scale consists of 17 items rated on a 5‐point Likert scale, ranging from 1 (strongly disagree) to 5 (strongly agree). Of these 17 items, 9 confirm breastfeeding, whereas 8 confirm formula feeding. Items regarding formula feeding are reverse‐scored. The total attitude score ranges from 17 to 85, with higher scores indicating more positive attitudes towards breastfeeding. Total IIFAS scores can be further categorized into the following groups: (1) positive attitude towards breastfeeding (IIFAS score of 70–85), (2) neutral attitude (IIFAS score of 49–69) and (3) positive attitude towards formula‐feeding (IIFAS score of 17–48) (Eksioğlu et al., 2016).

### 2.4 CFI

The CFI was developed for infants aged 6–12 months based on the existing CF recommendations for this age group. This index is scored using six variables: continued breastfeeding; avoiding bottle feeding; timely initiation of CF; dietary diversity, measured by the total number of food groups the infant consumed during the past 24 h; meal frequency (past 24 h); and food frequency, the frequency of food groups consumed by the infant during the first 7 days. The consumption of the following food groups is considered during measurements: cereals; pulses; milk and dairy products; meat and egg; fruits and vegetables rich in vitamin A; other fruits and vegetables; and foods prepared with oil, fat and butter (Garg & Chadha, 2009).

CFI analyses adherence to WHO's recommended standards for feeding practices. The CFI is determined by assigning a score of 0 for a potentially harmful practice and a score of 2 for a positive practice. Those in between these two practices are assigned a score of 1. The practices are considered positive or negative based on the WHO guidelines on CF for breastfed children (Pan American Health Organization, 2003). Regarding the food‐frequency score, each food group was scored individually, and the individual scores were summed to derive the final food‐frequency score. Thus, the CFI scores vary from a minimum of 3 to a maximum of 23 and are grouped into terciles to form three categories of CF practices: low, ≤6; medium, 7–16; and high, 17–23 (Garg & Chadha, 2009) (Table 1).

**Table 1:** Complementary feeding index variables and scoring patterns for complementary feeding practices.

Parameters	Variables	Scoring
Continued breastfeeding	Breastfeeding	No (0) Yes (2)
Bottle feeding	Uses bottles	No (1) Yes (2)
Timely initiation of complementary feeding	Complementary feeding was initiated on the completion of 6 months	No (0) Yes (2)
Dietary diversity (past 24 h)	For infants (6–8 months) Sum of cereals (grains/tubers) + pulses + milk (other than breastmilk) + GLVs and vitamin A‐rich fruits + egg + others^a^	0 (0) 1–2 (1) 3+ (2)
	For infants (9–12 months) Sum of cereals (grains/tubers) + pulses + milk (other than breastmilk) + GLVs and vitamin A‐rich fruits + egg + others	0 (0) 1–3 (1) 4+ (2)
Food frequency (past 7 days)	Starchy staples (grains/tubers)	0 (0) 1–3 (1) 4+ (2)
	Pulses	0 (0) 1–3 (1) 4+ (2)
	Milk (other than breastmilk)	0 (0) 1–3 (1) 4+ (2)
	Meat/eggs	0 (0) 1–3 (1) 4+ (2)
	Vitamin A‐rich fruits and vegetables	0 (0) 1–3 (1) 4+ (2)
	Other fruits/vegetables	0 (0) 1–3 (1) 4+ (2)
	Foods made with oil, fat or butter	0 (0) 1–3 (1) 4+ (2)
	Food frequency score=sum of scores for starchy staples + pulses + milk + meat/egg + vitamin A‐rich fruits/vegetables + foods made with fat	
Meal frequency (past 24 h)	For infants (6–8 months) No. of times the child was fed in the past 24 h	0 (0) 1 (1) 2+ (2)
	For infants (9–12 months) No. of times the child was fed in the past 24 h	0 (0) 1–2 (1) 3+ (2)

Abbreviations: CFI, Complementary feeding index; GLVs, Green‐leafy vegetables.

a Others include fruits, other seasonal vegetables, fat and sugar food groups.

### 2.5 Statistical analysis

Data were analysed using Statistical Package for Social Sciences software (version 20.0). WAZ, LAZ and WLZ were calculated using WHO Anthro software (2009) (WHO, 2009). For further analysis, the nutritional status of infants was classified according to the WHO criteria (WHO, 2006).

Furthermore, the infants were divided into two age groups: 6–8 and 9–12 months. A *p*‐value of <0.05 was considered to indicate statistical significance. All data were assessed for normality of distribution using the Kolmogorov–Smirnov test. Descriptive statistical data were presented using number, percentage, mean, and standard deviation. As continuous variables were normally distributed, comparisons between two groups were performed using the independent samples t‐test, whereas those among three or more groups were conducted using one‐way analysis of variance. When comparisons involved more than two groups, the Tukey test was used to determine the group from which the difference originated. The effect of CFI and IIFAS scores was analysed using multiple linear regression analysis for dependent variables WAZ and WLZ. The infant's gender, age, birth weight and different types of delivery, such as caesarean section or normal delivery, are among the factors that related to the infant's growth processes. In addition, breastfeeding plays a major role in the development of infants. In this context, feeding infants with breast milk or formula for the first 6 months or transitioning to complementary feeding within the first 6 months will also related to their growth processes. In the transition period to complementary feeding, the first food that the infant will meet or the consistency of the food to be given during this period are among the factors that determine the rate of weight gain of the infant. For these reasons, the model includes the following independent variables: infant sex, birth weight, infant age, mode of delivery, breastfeeding status for the first 6 months, formula feeding status for the first 6 months, CF status for the first 6 months, fruit feeding status for the first 3 days, consistency of complementary foods, CFI and infant feeding attitude scores.

### 2.6 Ethics

The study received ethics approval for publication from Bahçeşehir University Publication Ethics Committee (no. E‐20021704‐604.02.02‐57388).

## 3 RESULTS

This study included 720 infants, of whom 53.3% were female and 39.9% had a birth weight of 2501–3100 g. Approximately 33.3% of the infants were slightly overweight or obese based on WAZ scores, whereas 14.7% of them were underweight or malnourished. The mothers had a mean IIFAS score of 61.97 ± 7.63, and 79.4% of them had a neutral attitude towards infant feeding. They had a mean CFI score of 13.72 ± 4.24, with 67.8% of them being in the medium score category (Table 2).

**Table 2:** Characteristics of infants and mothers.

Parameters	6–8 months (*n* = 289)	9–12 months (*n* = 431)	All Infants (*n* = 720)
Sex			
Female	185 (64.0)	199 (46.2)	384 (53.3)
Male	104 (36.0)	232 (53.8)	336 (46.7)
Birth weight			
≤2500 g	16 (5.5)	23 (5.3)	39 (5.4)
2501–3100 g	100 (34.6)	187 (43.4)	287 (39.9)
3101–3500 g	121 (41.9)	138 (32.0)	259 (36.0)
3501–4400 g	48 (16.6)	68 (15.8)	116 (16.1)
≥4401 g	4 (1.4)	15 (3.5)	19 (2.6)
Mode of delivery			
Caesarean section	172 (59.5)	253 (58.7)	425 (59.0)
Vaginal	117 (40.5)	178 (41.3)	295 (41.0)
Breastfeeding for the first 6 months			
Yes	206 (71.3)	308 (71.5)	514 (71.4)
Formula feeding for the first 6 months			
Yes	115 (39.8)	153 (35.5)	268 (37.2)
Complementary feeding for the first 6 months			
Yes	98 (33.9)	119 (27.6)	217 (30.1)
Fruit feeding for the first 3 days			
Yes	178 (61.6)	258 (59.9)	436 (60.6)
Consistency of the complementary food			
Pureed	222 (76.8)	349 (81.0)	571 (79.3)
Liquid	67 (23.2)	82 (19.0)	149 (20.7)
Weight‐for‐age z‐score	0.07 ± 1.55	0.64 ± 1.37	0.42 ± 1.47
Weight‐for‐age category			
Malnourished	26 (9.0)	13 (3.0)	39 (5.4)
Underweight	40 (13.8)	27 (6.3)	67 (9.3)
Normal	150 (51.9)	224 (52.0)	374 (51.9)
Slightly overweight	47 (16.3)	108 (25.1)	155 (21.5)
Obese	26 (9.0)	59 (13.7)	85 (11.8)
Length‐for‐age z‐score	0.21 ± 2.30	0.39 ± 2.18	0.32 ± 2.23
Length‐for‐age category			
Long	61 (21.1)	84 (19.5)	145 (20.1)
Normal	178 (61.6)	288 (66.8)	466 (64.7)
Stunting	50 (17.3)	59 (13.7)	109 (15.1)
Weight‐for‐length z‐score	0.00 ± 1.67	0.72 ± 1.82	0.43 ± 1.80
Weight‐for‐length category			
Wasting	31 (10.7)	20 (4.6)	51 (7.1)
Underweight	50 (17.3)	42 (9.7)	92 (12.8)
Normal	131 (45.3)	195 (45.2)	326 (45.3)
Slightly overweight	47 (16.3)	84 (19.5)	131 (18.2)
Obese	30 (10.4)	90 (20.9)	120 (16.7)
Infant feeding attitude score	61.25 ± 7.99	62.45 ± 7.35	61.97 ± 7.63
Infant feeding attitude category			
Positive towards formula‐feeding	13 (4.5)	15 (3.5)	28 (3.9)
Neutral	235 (81.3)	337 (78.2)	572 (79.4)
Positive towards breastfeeding	41 (14.2)	79 (18.3)	120 (16.7)
Complementary feeding index score	12.08 ± 4.39	14.82 ± 3.76	13.72 ± 4.24
Complementary feeding index category			
Low	31 (10.7)	0 (0.0)	31 (4.3)
Medium	209 (72.3)	279 (64.7)	488 (67.8)
High	49 (17.0)	152 (35.3)	201 (27.9)

*Note*: Continuous variables are expressed as mean ± standard deviation, and categorical variables are expressed as percentages.

The infants were analysed for nutritional status in relation to maternal attitudes towards breastfeeding and CF. The WAZ scores were significantly higher in infants who received pureed foods than in those who received liquid food (0.49 ± 1.48 vs. 0.12 ± 1.40; *p* = 0.006). However, the nutritional status of infants did not vary by breastfeeding status for the first 6 months, formula feeding status for the first 6 months, CF status for the first 6 months and fruit feeding status for the first 3 days (all *p* > 0.05) (Table 3).

**Table 3:** Infants' nutritional status compared with maternal attitudes towards breastfeeding and complementary feeding.

		Weight‐for‐age z‐score	*p*	Weight‐for‐length z‐score	*p*
Breastfeeding for the first 6 months	Yes	0.43 ± 1.48	0.716	0.41 ± 1.84	0.579
	No	0.38 ± 1.45		0.49 ± 1.67	
Formula feeding for the first 6 months	Yes	0.39 ± 1.46	0.779	0.42 ± 1.79	0.931
	No	0.43 ± 1.48		0.43 ± 1.80	
Complementary feeding for the first 6 months	Yes	0.28 ± 1.64	0.112	0.27 ± 1.91	0.143
	No	0.47 ± 1.39		0.50 ± 1.74	
Fruit feeding for the first 3 days	Yes	0.44 ± 1.53	0.621	0.35 ± 1.84	0.164
	No	0.38 ± 1.37		0.55 ± 1.71	
Consistency of the complementary food	Pureed	0.49 ± 1.48	**0.006**	0.47 ± 1.80	0.195
	Liquid	0.12 ± 1.40		0.26 ± 1.78	

*Note*: Statistically significant values (*p* < 0.05) are shown in bold; Independent samples *t*‐test.

IIFAS and CFI scores of the mothers were analysed in relation to the nutritional status of infants. IIFAS scores were significantly lower among mothers of infants who were malnourished according to the WAZ category than among those of infants with normal weight. Likewise, IIFAS scores were lower among mothers of infants who were considered wasted (severe malnutrition) according to the WLZ category than among those of normal infants. Further, IIFAS scores were higher among mothers of slightly overweight infants than among those of obese infants in both categories (*p* < 0.05). CFI scores were lower among mothers of infants who were underweight in the WAZ category than among those of normal, mildly overweight and obese infants, whereas mothers of infants who were obese in the WLZ category had higher scores than those of underweight and normal infants (*p* < 0.05) (Table 4).

**Table 4:** Maternal attitudes towards infant feeding and complementary feeding indexes compared with infants' nutritional status.

	Weight‐for‐age category					Weight‐for‐length category				
	Malnourished	Underweight	Normal	Slightly overweight	Obese	Wasting	Underweight	Normal	Slightly overweight	Obese
Infant feeding attitude score^a^	57.76 ± 7.38	60.05 ± 8.90	62.49 ± 7.39	62.71 ± 7.52	61.78 ± 7.22	57.82 ± 7.84	61.11 ± 7.89	62.78 ± 7.68	62.42 ± 7.55	61.7 ± 6.73
*p*	**0.001**					**0.001**				
Complementary feeding index score^b^	12.23 ± 4.23	12.16 ± 3.99	13.89 ± 4.11	13.97 ± 4.23	14.47 ± 4.65	13.37 ± 3.70	12.60 ± 4.46	13.69 ± 4.15	13.58 ± 3.99	14.99 ± 4.55
*p*	**0.001**					**0.001**				

*Note*: Statistically significant values (*p* < 0.05) are shown in bold; One‐way ANOVA.

a Weight‐for‐age categories: Significant differences between malnourished and normal infants as well as between slightly overweight and obese infants. Weight‐for‐length categories: Significant differences between wasted and normal infants as well as between slightly overweight and obese infants.

b Weight‐for‐age categories: Significant differences between malnourished and obese infants; between underweight and normal infants; and between slightly overweight and obese infants. Weight‐for‐length categories: Significant differences between obese and underweight and normal infants.

**Figure 1:**
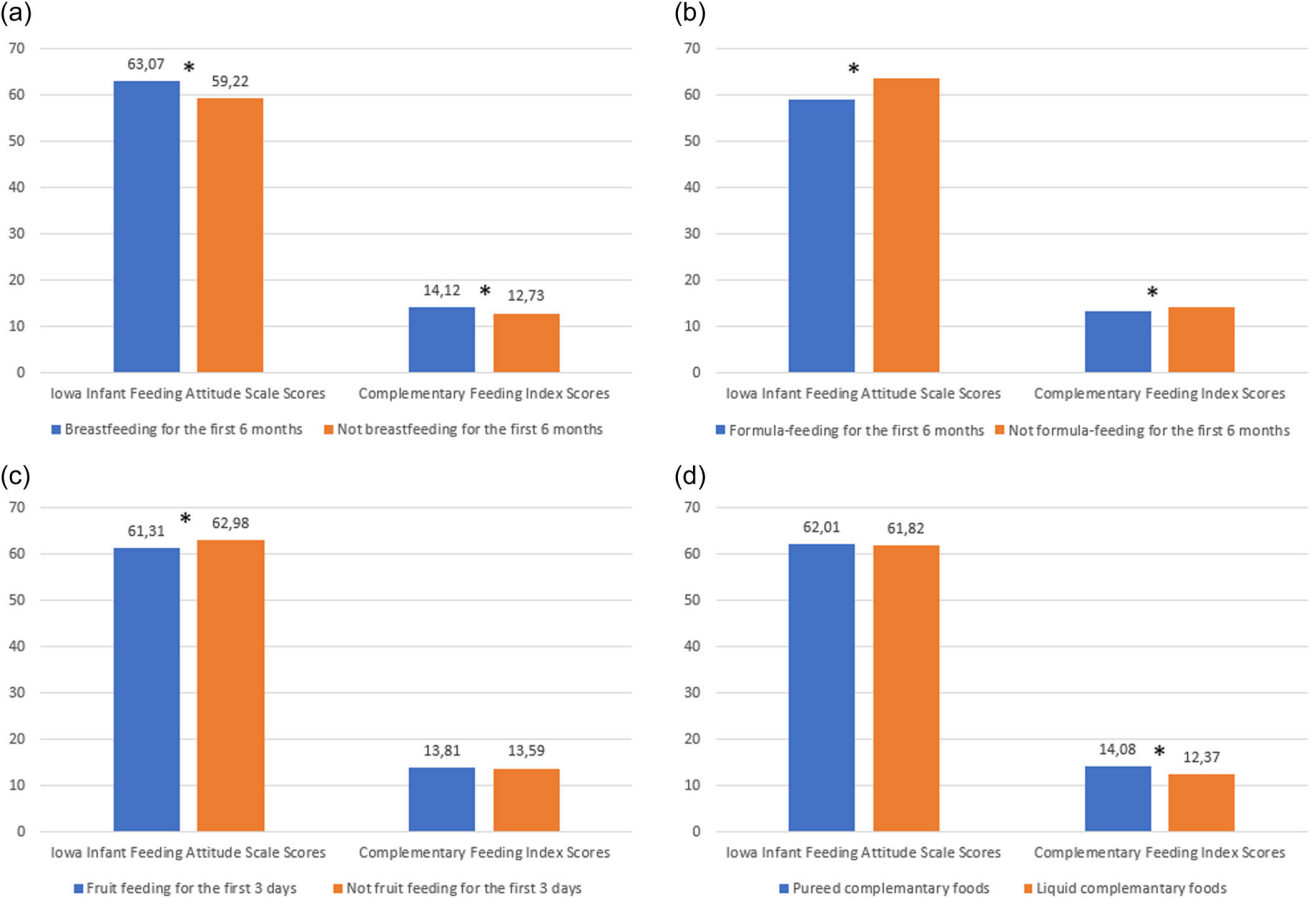
Association of maternal attitudes towards infant feeding and complementary feeding index with feeding practices. Infant feeding attitude scale and complementary feeding index scores were evaluated according to the following: (a) Breastfeeding for the first 6 months, (b) Formula feeding for the first 6 months, (c) Fruit feeding for the first 3 days and (d). Consistency of complementary food. *Statistical significance, *p* < 0.05.

This study investigated how mothers' IIFAS and CFI scores indicated infant feeding practices. IIFAS and CFI scores were significantly higher among mothers of infants who were breastfed for the first 6 months and significantly lower among mothers of infants who received formulas for the first 6 months. IIFAS scores were lower among mothers of infants who received fruit during the first 3 days than among those of infants who did not receive fruit. Further, CFI scores were higher among mothers of infants who received pureed foods than among those of infants who received liquid foods (*p* < 0.05) (Figure 1).

The association of selected maternal and infant characteristics with infants' nutritional status was analysed using multivariate regression analysis. Both models that used WAZ (R^2^ = 0.083, adjusted R^2^ = 0.064, F = 4.534, *p* = 0.001) and WLZ (R^2^ = 0.077, adjusted R^2^ = 0.059, F = 4.213, *p* = 0.001) as dependent variables yielded significant results. The independent variables included in the model were as follows: infant age, sex, birth weight, mode of delivery, breastfeeding status for the first 6 months, formula feeding status for the first 6 months, CF status for the first 6 months, fruit feeding status for the first 3 days, consistency of complementary foods and mothers' CFI and IIFAS scores. In the first model (WAZ scores), an infant age of 9–12 months (β = 0.524, *p* = 0.000), fruit feeding status for the first 3 days (β = 0.079, *p* = 0.016) and mothers' IIFAS score (β = 0.102, *p* = 0.008) were positively and significantly associated with infants' nutritional status, whereas delivery via caesarean section (C/S) was negatively and significantly associated (β = −0.365, *p* = 0.001) with infants' nutritional status. In the second model (WLZ scores), an infant age of 9–12 months (β = 0.681, *p* = 0.000) and mothers' CFI score (β = 0.075, *p* = 0.041) were positively and significantly associated with infants' nutritional status, whereas male sex (β = −0.444, *p* = 0.001) and delivery via C/S (β = −0.330, *p* = 0.014) were negatively and significantly associated with infants' nutritional status. Notably, the effect of mothers' IIFAS score (β = 0.098, *p* = 0.011) in the first model and CFI score (β = 0.070, *p* = 0.048) in the second model remained significant even after adjusting for confounding variables (Table 5).

**Table 5:** Regression analysis of predictors of nutritional status.

	Weight‐for‐age z‐score		Weight‐for‐length z‐score	
	β (95% CI)	*p*	β (95% CI)	*p*
Sex				
Female	1.00		1.00	
Male	−0.051 (−0.366−0.066)	0.173	−0.444 (−0.709–−0.179)	0.001
Birth weight				
≤2500 g	1.00		1.00	
2501–3100 g	−0.289 (−0.774–0.196)	0.242	−0.037 (−0.630–0.555)	0.902
3101–3500 g	−0.127 (−0.615–0.360)	0.608	0.027 (−0.568–0.623)	0.928
3501–4400 g	−0.386 (−0.911–0.139)	0.149	−0.199 (−0.839–0.442)	0.543
≥4401 g	−0.034 (−0.827–0.758)	0.933	0.048 (−0.919–1.014)	0.923
Infant age				
6–8 months	1.00		1.00	
9–12 months	0.524 (0.295–0.753)	0.000	0.681 (0.401–0.961)	0.000
Mode of delivery				
Caesarean section	−0.365 (−0.580–−0.150)	0.001	−0.330 (−0.592–−0.067)	0.014
Vaginal	1.00		1.00	
Breastfeeding for the first 6 months				
Yes	0.029 (−0.290–0.347)	0.861	−0.221 (−0.613–0.170)	0.268
No	1.00		1.00	
Formula feeding for the first 6 months				
Yes	0.041 (−0.259–0.341)	0.788	−0.005 (−0.377–0.367)	0.980
No	1.00		1.00	
Complementary feeding for the first 6 months				
Yes	−0.148 (−0.388–0.092)	0.227	−0.150 (−0.445–0.145)	0.317
No	1.00		1.00	
Fruit feeding for the first 3 days				
Yes	0.079 (−0.138–0.295)	0.016	−0.165 (−0.430–0.101)	0.224
No	1.00		1.00	
Consistency of the complementary food				
Pureed	0.291 (0.026–0.557)	0.031	0.131 (−0.94–0.458)	0.428
Liquid	1.00		1.00	
Complementary feeding index score	0.058 (−0.007–0.67)	0.149	0.075 (−0.001–0.085)	0.041
Infant feeding attitude score	0.102 (0.05–0.34)	0.008	0.060 (−0.004–0.32)	0.119
R² (adjusted R²)	0.083 (0.064)		0.077 (0.059)	
F value	4.534		4.213	
*p*‐value	**0.001**		**0.001**	

*Note*: Statistically significant values (*p* < 0.05) are shown in bold; Multiple linear regression.

## 4 DISCUSSION

The present study determined attitudes towards CF among mothers of infants aged 6–12 months as well as mothers'CFI and IIFAS scores and infants' WAZ, LAZ and WLZ scores. These indicators were analysed in terms of their association with CF practices and were compared with maternal attitudes towards infant feeding. In other words, the study investigated the basic principles of CF (consistency of food and suitability of food for age) and are related to some parameters. Analysis of the association between infants' intake of liquid or pureed foods and z‐scores revealed higher z‐scores among infants who were fed pureed foods, which is the ideal and recommended consistency for infants aged 6–9 months. However, unlike the consistency factor, the breastfeeding or formula feeding status and the first food item fed to the infant (vegetable puree and others) during the first 6 months had no association with z‐scores. Studies have shown that the training regarding CF given to parents affects the nutritional status of infants starting from the fourth month. These findings are in line with those of previous studies, which indicated that appropriate CF practices (e.g., consistency, timing and appropriate nutrients) significantly affect the z‐scores of infants and that infants fed with foods of appropriate consistency according to their age had higher z‐scores (Bergamini et al., 2022; Boswell, 2021).

This study revealed that the mode of delivery associated with WAZ and WLZ scores, and delivery via C/S are negatively associated with both WAZ and WLZ scores, which are parameters internationally accepted for defining malnutrition in infants. This finding is consistent with that of previous studies reporting that delivery via C/S leads to inadequate growth and development in later periods regardless of low birth weight (Miyazawa et al., 2023; Wedlund et al., 2023). However, a study from Japan reported that 366 infants delivered via C/S did not have lower WAZ scores than those delivered via vaginal delivery and had optimal scores. Their study concluded that C/S did not adversely affect the growth and development of infants (Miyayama et al., 2023). C/S has attracted attention as a perinatal factor involved in the pathogenesis of childhood diseases. Infants delivered via C/S have different gut microbiota compared with that of infants born via vaginal delivery due to differences in intrapartum bacterial exposure, and this difference may contribute to the development of obesity and immune‐related diseases in infants.

The WAZ score was found to be related to CFI and IIFAS scores. These scores were found to be significantly lower among mothers of malnourished infants than among other groups. Our study revealed that mothers with high IIFAS scores had infants with optimal WAZ scores. Subparameters of the IIFAS seem to support breastfeeding. There is a high level of evidence that breastfeeding provides more physiological and cognitive benefits than formula feeding. Thus, mothers who are aware of the benefits of breast milk are expected to have higher IIFAS scores. Another study reported significantly higher IIFAS scores among mothers who supported breastfeeding than among those who had a negative attitude towards breastfeeding (Twells et al., 2016). The IIFAS results in the present study can be confirmed by the lower attitude scores found among mothers of infants who received formula feeding for the first 6 months.

Although the total IIFAS score did not predict the CF status of the infant, WAZ scores were significantly higher in infants of mothers with high IIFAS scores than in infants of mothers with low IIFAS scores. In our study, we found that mothers of infants with a healthier weight were more likely to have a positive attitude towards breastfeeding and there are studies in the literature supporting this result (Atik, 2021; Shosha, 2015). Moreover, Zongrone et al. (2012) showed that exclusive breastfeeding was protective against wasting. Further, the age‐appropriate introduction of CF or families' nutritional attitudes had a positive linear association with the WAZ score (Boucheron et al., 2020; Wood et al., 2021). This indicates that mothers who believe that breastfeeding is more convenient, healthier and cheaper are more likely to initiate CF at the appropriate time and may achieve better dietary diversity.

WAZ scores were found to be significantly higher among infants of mothers with high CFI scores than among those of mothers with low CFI scores. More effective adherence to WHO CF guidelines was related to more positive weight results. Consistent with our study findings, a systematic meta‐analysis (*n* = 16) revealed that the level of mothers' knowledge about CF positively affects WAZ scores in infants (Lassi et al., 2013). Similarly, another study reported that CF training had positive effects on the nutritional status of infants in terms of weight and height (Imdad et al., 2011). These reports are consistent with our study, revealing that the CFI score and infant age were associated with WLZ scores (Ma et al., 2012; Rahman et al., 2022; Twabi et al., 2021). Indeed, families' level of knowledge and attitudes towards nutrition may facilitate infant growth. Evidently, most infants undergoing weaning do not consume a balanced diet, mainly due to a lack of mothers' knowledge.

### 4.1 Strengths and Limitations

The strength of this study is derived from the fact that it is probably the first study in Turkey to investigate the effect of maternal attitudes towards feeding on the nutritional status of infants using IIFAS and CFI scores. Another strength is the use of previously validated questionnaires. Despite the promising results of this research, it has several limitations. Our regression models were able to explain approximately 6% of the nutritional status of infants. This suggests that other factors are not included in our model, such as genetic factors, socioeconomic and educational status of the family and parental overweight or obesity status, which may affect nutritional status. The cross‐sectional nature of the study limits the analysis to investigating correlations rather than determining causality. Another limitation is the calculation of z‐scores based on the height and weight measurements reported by the mothers. This limitation includes some concerns such as misreporting of measurements by mothers. Moreover, although the results can be applied to other regions similar to Turkey, it is not possible to generalize the results of this single‐country study to all developing countries.

## 5 CONCLUSIONS

Studies have reported that the introduction of CF at an appropriate time, with optimal nutrient composition and consistency, has a positive effect on infant health outcomes. Our findings are in line with the reports indicating that mothers' level of knowledge about CF and appropriate CF practices has a significant effect on the nutritional status of infants. Considering the impact of growth and developmental delay on the occurrence of chronic diseases, it is important to provide optimal CF training to expectant mothers by health personnel even during the pregestational period. In conclusion, we believe that the tools used in the present study can be incorporated into public health programmes to address issues related to CF in a comprehensive manner and monitor changes in feeding practices. CFI can be used as an easy tool by clinicians for identifying, targeting and monitoring the deficient CF practices. In line with the findings of our study, the use of CFI in infants can be made widespread as a nutrition screening tool.

## AUTHOR CONTRIBUTIONS

Bilge Meral Koc was responsible for data collection, methodology, manuscript writing and editing. Tugce Ozlu Karahan was responsible for methodology, manuscript writing and editing. Ezgi Arslan Yuksel was responsible for data collection, methodology, manuscript writing. Gokcen Garipoglu conceptualized the manuscript.

## CONFLICT OF INTEREST STATEMENT

The authors declare no conflict of interest.

## Data Availability

The data that support the findings of this study are available from the corresponding author upon reasonable request.
